# Supplementary material for: The Pupa Stage Is the Most Sensitive to Hypoxia in Drosophila melanogaster
Source: Int J Mol Sci. 2024 Jan 5;25(2):710. doi: 10.3390/ijms25020710 (PMC10815303; doi:10.3390/ijms25020710)
Supplement: Supplementary file 1 [file ijms-25-00710-s001.zip › ijms-2794524-supplementary.pdf]

Figure S1

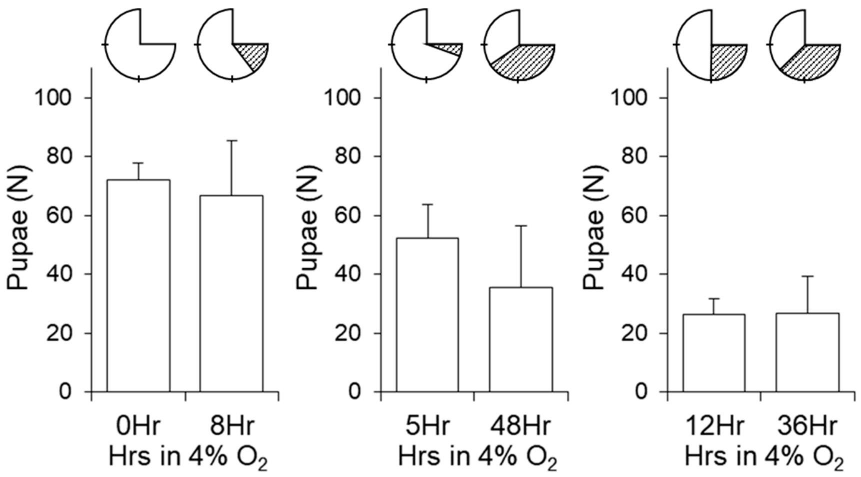

Figure S1. Pupation of the embryos of control fly line, *w1118*, when exposed to 4% O_2 for different time-period. The number of pupae (N) were similar within the same batch irrespective of the 4% O_2 treatment duration. Top: The shaded part indicate the life-cycle stage and the time-period at which the flies were kept in 4% O_2 and the unshaded part is the time period in room air.

Figure S2

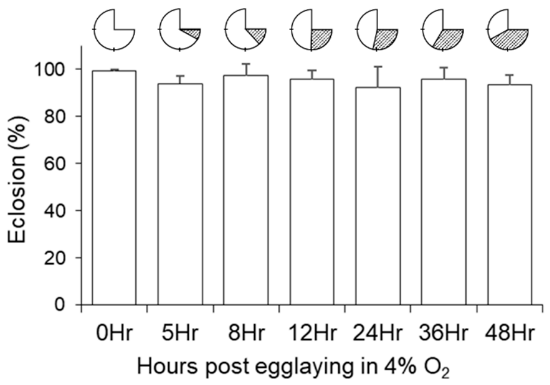

Figure S2. The eclosion rates for the *w1118* embryos treated with 4% O_2 for up to 48 h. The eclosion rates were >90% in all time points. Top: The shaded part indicate the life-cycle stage and the time-period at which the flies were kept in 4% O_2 and the unshaded part is the time period in room air.

Figure S3:

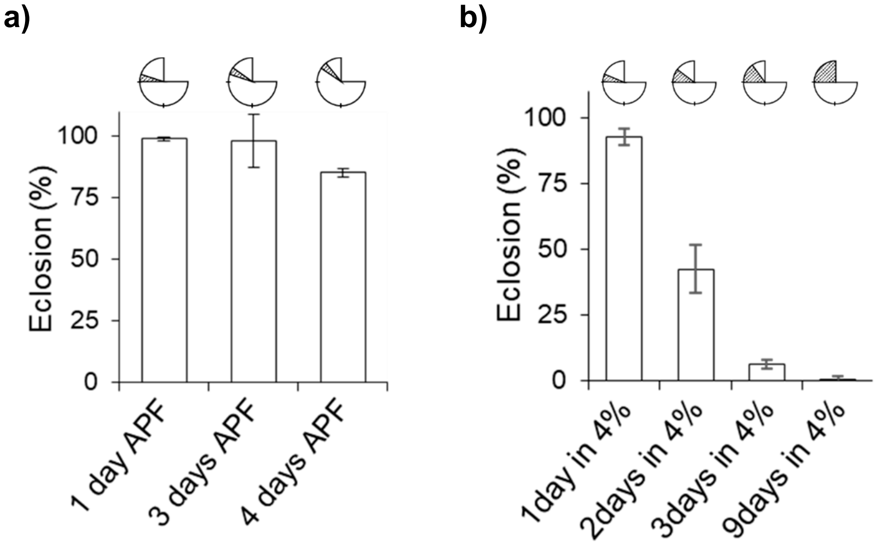

Figure S3. Two days exposure to 4% O_2 during pupae is the critical time duration. a) The eclosion rates of *w1118* pupae when exposed to 4% O_2 for 1 day at 1, 3 and 4 days after pupae formation (APF) were >85%. b) The eclosion rates of *w1118* when exposed to 4% O_2 for different time (days) period. Treating pupae of 1-3 days APF (mixed age) with 4% O_2 for 1, 2, 3 and 9 days were 93%, 42%, 6.1% and 0%, respectively. Top: The shaded part indicate the life-cycle stage and the time-period at which the flies were kept in 4% O_2 and the unshaded part is the time period in room air.

Table S1

Table S1. Eclosion rate (%) of *y^1v^1*, *w1118* and *HA-flies* in 3.5%, 3.7%, 4%, 5% and 21% O_2 environment.

	y^1v^1	***w1118***	***HA-flies***
**3.5% O_2**	0 (±0.0)	0 (±0.0)	53.9 (±7.9)*
**3.7% O_2**	0 (±0.0)	0 (±0.0)	80.2 (±3.9)*
**4.0% O_2**	1.7 (±1.6)	3.8 (±0.9)	76.9 (±13.0)*
**5.0% O_2**	18.7 (±1.9)	11.1 (±6.0)	78.2 (±8.2)*
**21% O_2**	99.6 (±0.8)	100.0 (±0.0)	99.5 (±0.8)

*, p-value vs *y^1v^1* and *w1118* < 0.005.

Table S2

Table S2. The p-values of eclosion rate comparisons of fly lines kept in different O_2 environments. The values depicts the comparisons between the same fly line kept in different O_2, i.e., between the O_2 level depicted in rows vs the O_2 level depicted in column.

		y^1v^1				***w1118***				***HA-flies***			
	**O_2 level**	**3.5%**	**3.7%**	**4.0%**	**5.0%**	**3.5%**	**3.7%**	**4.0%**	**5.0%**	**3.5%**	**3.7%**	**4.0%**	**5.0%**
	**3.7%**	NA											
y^1v^1	**4.0%**	0.133	0.133										
	**5.0%**	0.0003	0.0003	0.002									
	**21%**	<0.0001	<0.0001	<0.0001	<0.0001								
	**3.7%**					NA							
w1118	**4.0%**					0.002	0.002						
	**5.0%**					0.033	0.033	0.1					
	**21%**					NA	NA	<0.0001	<0.0001				
	**3.7%**									0.007			
HA-flies	**4.0%**									0.059	0.701		
	**5.0%**									0.021	0.725	0.893	
	**21%**									0.0006	0.001	0.0402	0.012

Table S3

Table S3. The eclosion rate of y^1v^1 and *w1118* when the flies were exposed to 4% O_2 environment.

	Embryo	Larvae			Pupae			% Eclosion	
**Hypoxia exposure stage**	**embryo**	**1st instar**	**2nd instar**	**3rd instar**	**early**	**mid**	**late**	y^1v^1	***w1118***
3 days APF	4	4	4	4	4	4	21	66.23 ±8.72	54.35 ±11.33
2 days APF	4	4	4	4	4	21	21	88.98 ±7.04	66.14 ±22.09
1 day APF	4	4	4	4	21	21	21	100.0 ±0.00	99.56 ±0.76
3rd instar	4	4	4	21	21	21	21	100.0 ±0.00	100.0 ±0.00
2nd instar	4	4	21	21	21	21	21	100.0 ±0.00	100.0 ±0.00
1st instar	4	21	21	21	21	21	21	98.08 ±1.92	97.55 ±2.54
All normoxia	21	21	21	21	21	21	21	100.0 ±0.00	100.0 ±0.00
at 3 days APF	21	21	21	21	21	21	4	0.00 ±0.00	0.00 ±0.00
at 2 days APF	21	21	21	21	21	4	4	0.00 ±0.00	0.00 ±0.00
at 1 day APF	21	21	21	21	4	4	4	0.00 ±0.00	0.00 ±0.00
at 1st instar	21	4	4	4	4	4	4	2.34 ±2.09	0.52 ±0.90
All hypoxia	4	4	4	4	4	4	4	1.67 ±2.89	0.00 ±0.00

4 and 21 indicated the O_2 levels at which the flies were kept.

Table S4

Table S4. Eclosion rate (%) of pupae at different days APF exposed to 2 days of 4% O_2.

	Eclosion rate (%)
**Normoxia**	100.00 ±0.00
**1 Day APF**	89.28 ±5.37
**2 Days APF**	0.00 ±0.00
**3 Days APF**	0.00 ±0.00
**4 Days APF**	35.07 ±6.76
**5 Days APF**	51.33 ±8.08

APF, after pupae formation.

Table S5

Table S5. Eclosion rate (%) of pupae at different days APF exposed to 3 days of 4% O_2.

	Eclosion rate (%)
**Normoxia**	100.00 ±0.00
**1 Day APF**	81.25 ±12.70
**2 Days APF**	12.58 ±8.44 *
**3 Days APF**	23.61 ±1.60 *

APF, after pupae formation; p-value (vs 1 Day APF) < 0.001.
